# Neglected Patellar Tendon Rupture Reconstruction With Peroneus Longus Tendon Autograft and Modified Quadriceps Lengthening: A Case Report

**DOI:** 10.5435/JAAOSGlobal-D-24-00107

**Published:** 2024-10-09

**Authors:** Aditya Fuad Robby Triangga, Anak Agung Ngurah Nata Baskara, Prisilla Desfiandi, A. Faiz Huwaidi

**Affiliations:** From the Department of Orthopedics and Traumatology (Mr. Triangga, Mr. Nata Baskara, Dr. Desfiandi), Faculty of Medicine, Public Health and Nursing, Universitas Gadjah Mada/RSUP, Dr. Sardjito Hospital, D.I. Yogyakarta, Indonesia, and the Faculty of Medicine (Mr. Triangga, Mr. Nata Baskara, Dr. Desfiandi, Dr. Huwaidi), Public Health and Nursing, Universitas Gadjah Mada, D.I. Yogyakarta, Indonesia.

## Abstract

Neglected patellar tendon reconstruction poses several challenges, including considerations for graft options, quadriceps lengthening, and postoperative extensor strength. There is no universal consensus on the optimal technique for neglected patellar tendon reconstruction. In this study, we report the patient with a neglected patellar tendon resulting from trauma occurring 1 year earlier. This patient has ambulatory disturbance, a high-riding patella, and notable limitations in knee range of motion. Our reconstruction procedure involves the use of the peroneus longus autograft, in conjunction with prolene mesh and modified quadriceps tendon lengthening. The results demonstrate an adequately restored patellar position, as evidenced by an Insall-Salvati ratio of 1.3. The patient exhibits a good functional outcome.

Compared with quadriceps tendon rupture or patellar fracture, patellar tendons are not as frequently seen in the extensor mechanism of the lower limb.^1^ Cases of neglected patellar tendon rupture are even rarer and pose challenges for surgeons. Unlike acute patellar tendon rupture that can be repaired immediately, neglected cases require tendon augmentation and quadriceps lengthening procedures.^2-4^ This is attributed to scarring, proximal patellar retraction, atrophy of the surrounding connective tissue, lack of viable tissue, and patella alta, which complicate the reconstruction procedure.^2^

There is no universal consensus regarding the graft choice and method for quadriceps lengthening in patellar tendon reconstruction, especially in chronic cases.^2^ However, most studies use a hamstring graft, quadriceps turndown flap, free fascia lata graft, Achilles tendon graft, contralateral bone patellar, and even synthetic tendons such as ligament advanced reinforcement system.^5^ The utilization of the peroneus longus tendon as a graft in patellar reconstruction procedures is rarely reported. In this case report, we present patellar tendon reconstruction achieved through single reconstruction after quadriceps lengthening with the V-Y technique combined with the pie-crusting technique, which was augmented by prolene mesh and the peroneus longus autograft.

## Case Report

A 47-year-old male farmer presented to our hospital with a 12-month report of right knee pain, difficulty in extending the knee, and ambulatory limitations. The patient admitted that he fell with his knee flexed 1 year earlier, which impacted his infrapatellar. After the incident, the patient initially sought medical attention at a primary health facility, where he received an injection of painkillers. The patient did not seek additional medical care.

On physical examination, a high-riding patella of the right knee was observed. Range-of-movement examination revealed 0° passive extensions and 120° on passive flexion, with an active movement of 60° to 90°. When palpated, a gap was identified between the patella tendon and tibial tuberosity.

**Figure 1:**
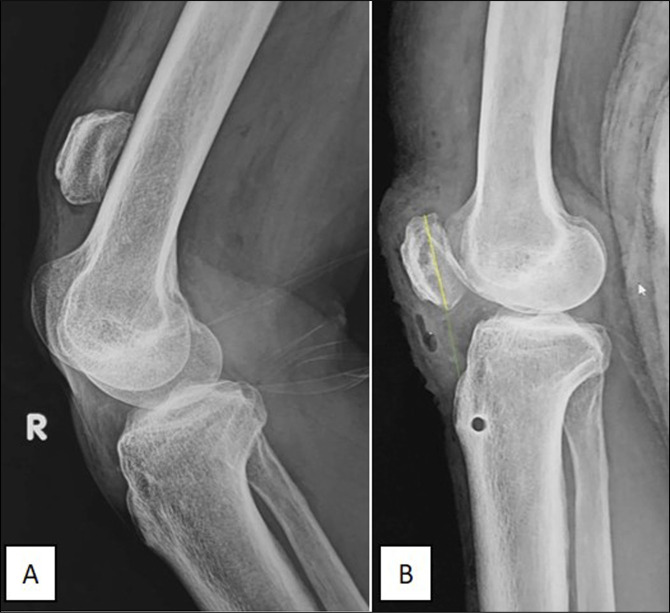
Plain radiographs of the right knee. **A,** Preoperative knee radiograph showing patella alta (**B**). Postoperative knee radiograph showing the patella in its anatomical position.

Plain radiograph examination with lateral projections showed patella alta with an Insall-Salvati ratio of more than 1.2 (Figure 1, A). Based on these findings, we diagnosed the patient with neglected complete patellar tendon rupture. The patient agreed to undergo a surgical intervention.

### Preoperative Planning

We anticipated challenges in restoring the patella to its anatomical position due to fibrosis and scarring, along with the need for a quadriceps lengthening procedure. Although a V-Y quadricepsplasty was initially planned, concerns about progressive scarring prompted the decision to combine it with a pie-crusting technique on the quadriceps tendon. To address concerns about the potential strength reduction in the quadriceps muscles after V-Y quadriceplasty, an augmentation procedure was performed using a peroneus longus tendon autograft supplemented by synthetic prolene mesh. The objective was to provide the required length of quadriceps for patella repositioning while preserving optimal extensor strength.

### Positioning

The patient was positioned supine on the operating table under general anesthesia. A tourniquet was used. The right leg was prepared and draped for the procedure.

## Harvesting of the Peroneus Longus Tendon

We marked the incision area after identifying the fibular head, followed by blunt dissection to isolate the peroneus longus from the surrounding tissue. An additional incision, approximately 3 cm in length, was made above the lateral malleolus to identify the peroneus longus tendon. Using a closed tendon stripper, the peroneus longus tendon was harvested, leaving approximately 5 cm from the fibular head intact to prevent peroneal nerve injury.

## Technique

**Figure 2:**
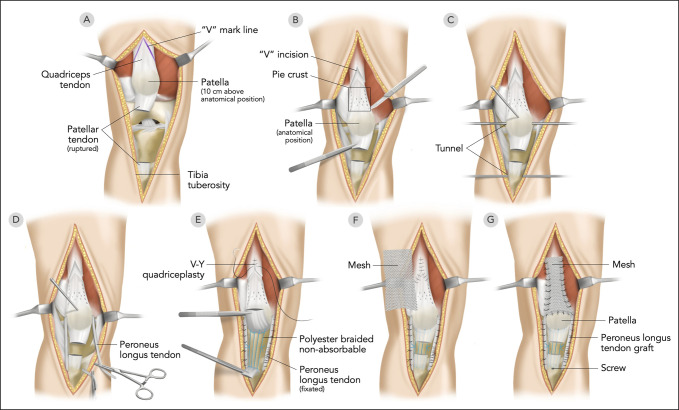
Original illustration describing our reconstruction process.

An anterior, midline, and longitudinal incision was made from the superior pole of the patella to the tibial tuberosity with the subsequent undermining of the skin and connective tissue. A 14-cm gap between the patella and its tendon was found after removing the necrotic tissue. To resume the reconstruction procedure in our case, we provide the original illustration of our reconstruction (Figure 2).

### Quadriceps Lengthening

We retracted the distal quadriceps tendon to achieve the anatomical position. We confirmed this position by measuring the Insall-Salvati ratio on the right knee of the patient. Subsequently, the leg was positioned to full extension, and we performed a V-Y quadriceplasty combined with the pie-crusting technique on the quadriceps tendon. The pie crust was created in 3 rows with a 1-mm distance, using an 18G needle.

### Quadriceps Augmentation

**Figure 3:**
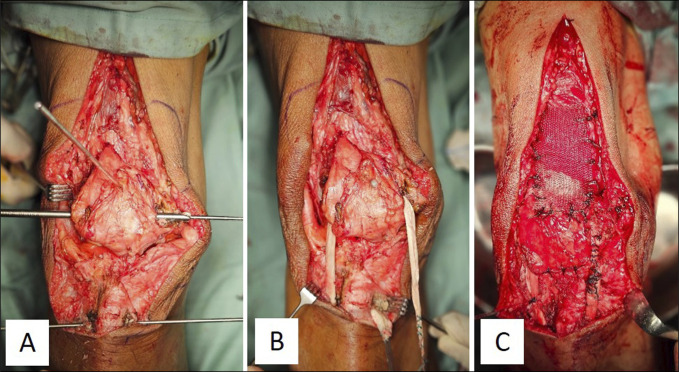
Images demonstrating our reconstruction procedure of quadriceps augmentation using peroneus longus tendon.

2 methods were used for augmentation. First, a previously obtained peroneus longus autograft was used. We created an 8-mm tunnel in the superior part of the patella and the tibial tuberosity. The peroneus longus tendon was inserted from the lateral to the medial side through the tunnel. Additional augmentation involved synthetic prolene mesh where the mesh was folded up to 8 times and shaped to a size of 3 × 15 cm, and the proximal mesh was sewn to the quadriceps tendon while the distal mesh was attached to the superior patella and sewn to the peroneus longus tendon (Figure 3). The peroneus longus tendon was fixed in the tibial tunnel using a bioabsorbable screw of the same diameter, similar to when we used the hamstring tendon. The mesh was then sutured into the surrounding tissue. After ensuring knee stability, the tourniquet was released, and the wound was closed using 2.0 Vicryl sutures.

### Postoperative Rehabilitation and Follow-Up

**Figure 4:**
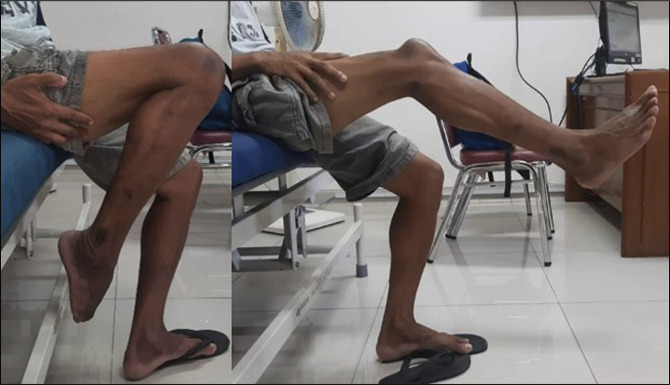
Images demonstrating the range of motion of the right knee after reconstruction. The active range of motion achieved is 105° to 20°.

We conducted a plain radiograph evaluation on day 1 after the reconstruction procedure (Figure 1, B). The patient's right limb was immobilized using a long leg splint, and he remained non–weight bearing for 4 weeks. Physical therapy started in the third week. At the 2-month follow-up, the patient achieved an active knee motion ranging from 105° to 20° with no difficulty in ambulation (Figure 4).

## Discussion

Unilateral patellar tendon rupture is primarily caused by trauma, leading to symptoms such as pain, extensor lag, and difficulty in walking. Owing to the morbidity, surgical intervention is the benchmark because nonsurgical approaches yield suboptimal outcomes.^6^ Notable differences were observed between acute and chronic patellar tendon reconstructions. Chronic cases involve addressing issues such as quadriceps contracture, scarring, soft-tissue damage, and patella alta.^3,4^ Consequently, procedures such as quadriceps lengthening and tendon graft utilization become crucial for restoring the anatomical patellar position and improving knee extensor function and structure.

Nowadays, there is no agreement regarding the best tendon graft for chronically ruptured patellar tendons. A systematic review conducted by William et al. in 2022 reported that the most commonly used tendons are the hamstring, followed by bone-patellar tendon-bone, direct repair, and Achilles tendon.^6^ A study highlighted its superior load capacity (1170.4 ± 203 N) from the peroneus longus tendon compared with the hamstrings or patellar tendon. The safety of peroneus longus is affirmed by its potential for grafting, maintenance of strength, biomechanical benefits, and assurance of safe leg elevation function.^7^ The peroneus longus muscle functions to evert the foot and maintain the arch for proper walking mechanics. The peroneus longus tendon also acts as a passive stabilizer of the ankle. However, studies have shown that harvesting the peroneus longus tendon does not negatively affect ankle function. By the sixth month after harvesting, ankle function is 100% normal and comparable with the nonharvested foot. This supports the safety of using the peroneus longus tendon as a graft source.^8^

Moreover, peroneus longus tendon offers a promising option in anterior cruciate ligament reconstruction, providing strength with a lower risk of donor site morbidity and comparable patient outcomes with hamstring grafts and maintaining stability, gait, and ankle movement.^9,10^ Although there have been reports on the use of peroneus longus tendon autografts for reconstructing the extensor mechanism associated with patellar fractures and for reconstructing patellar tendon ruptures after total knee arthroplasty procedures, our report contributes to the existing evidence.^10,11^ We present a case of reconstruction for a neglected patellar tendon rupture in a native knee without an accompanying patellar fracture and showed satisfactory results.

Reports of extensor lag and elongation in tendon rupture reconstructions with allografts have prompted the consideration for the use of synthetic materials such as prolene mesh.^11,12^ Prolene mesh acts as a pad for fibrous tissue growth and offers a mechanical advantage by providing tensile strength. This innovative approach has demonstrated an 84% reduction in extensor lag, with minimal foreign body reactions and cost effectiveness.^13,14^

Addressing the issue of quadriceps tendon shortening in chronic patellar tendon rupture reconstruction, common procedures such as V-Y quadriceplasty and the pie-crusting technique are used. While V-Y quadriceplasty requires longer immobilization, the pie-crusting technique facilitates early high-intensity activities by preserving tendon integrity.^15^ In our case, a combination of V-Y quadriceplasty and the pie-crusting technique was done and the patient's outcomes were successfully improved.

## Conclusion

The utilization of an autologous peroneus longus tendon graft, synthetic prolene mesh, and a quadriceps lengthening procedure combining V-Y quadriceplasty with the pie-crusting technique yielded satisfactory results in chronic patellar tendon rupture reconstruction. This approach seems effective and safe, but unfortunately only with a limited sample size. Future studies with a larger sample size and extended follow-up durations would prove the validity of our findings.
